# Influence of Amino Acids, Dietary Protein, and Physical Activity on Muscle Mass Development in Humans

**DOI:** 10.3390/nu5030852

**Published:** 2013-03-13

**Authors:** Kasper Dideriksen, Søren Reitelseder, Lars Holm

**Affiliations:** 1 Institute of Sports Medicine, Department of Orthopedic Surgery M81, Bispebjerg Hospital, Bispebjerg Bakke 23, DK-2400 Copenhagen NV, Denmark; E-Mails: s.reitelseder@gmail.com (S.R.); l.holm.isotope@gmail.com (L.H.); 2 Center for Healthy Aging, Faculty of Health and Medical Sciences, University of Copenhagen, Blegdamsvej 3, DK-2200 Copenhagen N, Denmark

**Keywords:** Protein, amino acids, muscle protein synthesis, muscle protein breakdown, resistance exercise

## Abstract

Ingestion of protein is crucial for maintenance of a variety of body functions and within the scope of this review we will specifically focus on the regulation of skeletal muscle mass. A quantitative limitation exists as to how much muscle protein the body can synthesize in response to protein intake. Ingestion of excess protein exerts an unwanted load to the body and therefore, it is important to find the least amount of protein that provides the maximal hypertrophic stimulus. Hence, research has focused on revealing the relationship between protein intake (dose) and its resulting stimulation of muscle protein synthesis (response). In addition to the protein amount, the protein digestibility and, hence, the availability of its constituent amino acids is decisive for the response. In this regard, recent studies have provided in-depth knowledge about the time-course of the muscle protein synthetic response dependent on the characteristics of the protein ingested. The effect of protein intake on muscle protein accretion can further be stimulated by prior exercise training. In the ageing population, physical training may counteract the development of “anabolic resistance” and restore the beneficial effect of protein feeding. Presently, our knowledge is based on measures obtained in standardized experimental settings or during long-term intervention periods. However, to improve coherence between these types of data and to further improve our knowledge of the effects of protein ingestion, other investigative approaches than those presently used are requested.

## 1. Introduction

The preservation and development of skeletal muscle mass is essential for maintenance of health and life quality. Due to its volume, the skeletal muscle makes up the primary site for disposal of nutrients and energy consumption in the body and, hence, plays an essential role for weight regulation. Therefore, skeletal muscle mass is important in protecting against the development of metabolic conditions such as obesity, hyperlipidemia, cardiovascular disease, and type II diabetes [1,2]. Furthermore, skeletal muscle makes up a huge storage of amino acids (AA), which, when recruited, are crucial for making acute phase proteins in the fight against critical illness or in wound healing following severe trauma [1]. Limiting the loss of skeletal muscle mass during periods of illness or injury is essential to decrease patient morbidity and increase recovery outcome [3]. 

In this review, we discuss protein intake with special emphasis on type and amount alone and in combination with exercise regimens that induce changes in muscle mass with the purpose to link findings from acute changes in muscle protein turnover to long-term changes in muscle mass. Finally, we will discuss in which directions future research in this field should focus.

## 2. Whole-Body Effects of Protein Intake

The daily requirement for dietary protein is defined as the minimum amount resulting in a whole-body net balance of zero. Since excessive protein intake adds load on the kidneys, proteins should not be consumed in overly amounts and should be a matter of concern, especially in elderly individuals [4]. Therefore, research to address the exact amount to meet the requirements for body remodeling is crucial. The protein requirement has been vigorously debated due to difficulties in specifying which parameter(s) (e.g., whole-body protein mass, muscle mass, physical function, immune function, or metabolic function) it should be based upon. Additionally, the protein requirement depends on quality—that is AA composition and digestibility—and physical activity level. A greater protein requirement has been reported in bodybuilders and endurance trained athletes compared to sedentary young men [5]. In a retrospective re-assessment of data from more than 100 resistance-trained men and women in the age of 50–80 year, a positive relationship was established between the dietary protein intake and the change in lean body mass (LBM) and a daily protein intake of 1.0 g/kg body weight (BW) was established as sufficient [6]. Whereas, in sedentary young and elderly men and women, a daily protein intake of 0.85 g/kg BW was found to be adequate [7], indicating that persons participating in resistance training may need a higher protein intake than sedentary persons. 

During periods of inactivity, increasing the level of protein intake may be particular relevant to diminish the loss of muscle mass. During 7 days of bed rest an enhanced daily protein intake (from 0.6 to 1.0 g/kg BW) resulted in a maintained whole-body protein synthesis and nitrogen loss in young men [8]. Whereas, an unchanged daily protein intake of 1.0 g/kg BW during 14 days of bed rest could not prevent a decline in the postabsorptive whole-body protein turnover rate in young men [9]. Collectively, it was hypothesized that an elevated protein intake is required to maintain whole-body postabsorptive protein turnover during inactivity [8,9]. Thus, when measured with the techniques usually applied on whole-body measurements, the increase in protein intake *per se* seems to be more important than the actual level of protein intake. The change in protein intake is crucial because the efficiency of protein utilization adapts to changes in physical activity level, energy balance, and amount of protein intake [10,11,12,13] and it takes time before these adaptation processes are complete [5,12,13,14]. This means that a constantly high protein intake does not necessarily lead to accumulation of body protein mass. 

## 3. Skeletal Muscle Effects of Protein Intake

**Figure 1:**
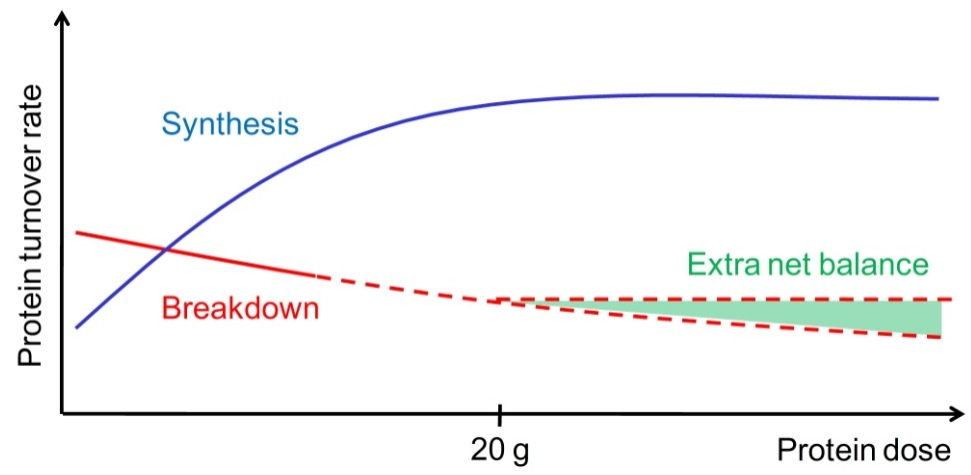
Illustration of muscle protein synthesis (MPS, blue curve) and muscle protein breakdown (MPB, red curve) rates in response to graded intake of protein. With increasing protein ingestion, MPS increases fast but reaches a plateau (at approximately 20 g high quality protein). In contrast to the MPS, MPB are hypothesized to decrease slightly but continuously with increasing protein or amino acid intakes. Therefore, the net muscle protein balance can possibly benefit from an even greater protein intake than known to stimulate MPS maximally (illustrated by the extra net balance area marked with green).

Moving from whole-body to specific tissues, alternative techniques are often used. Acknowledging that tissue-specific adaptations do exist, different methods can also explain why tissue-specific adaptations are not necessarily reflected by whole-body nitrogen balance and protein turnover measurements. Typically, tissue-specific protein turnover is measured by the direct-incorporation method, where only the synthesis rates are measured. Using this approach, skeletal muscle protein synthesis (MPS) rate is leveling off with increasing doses of protein intake (Figure 1). In support of that, no difference in MPS was found following 10 days of isocaloric diets containing 1.5 or 3.0 g protein/kg LBM/day in young and elderly men [15]. Additionally, within the acute perspective, no difference in myofibrillar MPS could be located between isocaloric low-protein (7 E%) or high-protein drinks (28 E%) in elderly men and women consumed every 30 min during 7.5 h [16]. Collectively, these results indicate that high-protein diets do not enhance MPS, as long as the requirements for energy and protein are fulfilled. Instead, strategies regarding the amount and type of protein feeding appear to be important for optimizing the muscle protein accretion.

### 3.1. The “Muscle Full” Concept

An elevation of the plasma AA concentration to almost twice the basal values leads to stimulation of myofibrillar MPS after 30–60 min of AA infusion, but the stimulation is only maintained for a period of ~90 min even though AA is continuously infused for 6 h [17]. Hence, muscle intracellular hyperaminoacidemia *per se* does not stimulate myofibrillar MPS continuously. More recently, myofibrillar MPS was shown to increase from 60 to 180 min after ingestion of 25 g whey protein [18]. Furthermore, the MPS response and the essential amino acid (EAA) concentration fainted simultaneously in that study [18]. However, it is possible that the MPS response would have declined earlier, had it been measured during shorter periods of time in the early postprandial period [18]. This statement is based on recent results showing that the myofibrillar MPS did increase 45–90 min after ingestion of 45 g of whey protein [19]. Moreover, the MPS response declined even though the EAA concentration and the intracellular anabolic signaling was still elevated [19]. Thus, the muscles become refractory to persistent elevations in the intramuscular AA concentration, irrespective of the mode of AA delivery. This phenomenon may be due to inhibitory signal mechanism, suggested to involve endoplasmic reticulum stress [19]. Recently, we showed that the myofibrillar MPS could be stimulated for as long as 8 h with continuous protein feeding in resting muscle [20]. Although, the stimulatory effect found by Bechshoeft *et al.* was markedly prolonged [20], the total amount of AA stored in the resting muscle was approximately similar to what was found by Bohe *et al.* and Atherton *et al.* [17,19]. The more intense stimulation of myofibrillar MPS reported by Bohe and Atherton is probably achieved by the more pronounced increase in circulating AA. Therefore, there seems to be a certain quantitative limitation to the absolute amount of AA that can be stored as contractile proteins in resting skeletal muscle during hyperaminoacidemia, a phenomenon termed the “muscle full” concept [19].

### 3.2. Protein Dose and Muscle Protein Synthesis

Amino acids exert a significant stimulatory effect on the MPS. While most AA are contained in proteins, it appears only to be the EAA that stimulate MPS and, thus, non-essential AA do not have any additional effect on MPS [21]. Specifically, the branched chain AA, leucine, has been shown to be a key regulator of anabolic signaling in human skeletal muscles [22,23]. However, although leucine is able to initiate the translational processes, the other EAA are probably necessary as well to induce a sustainable MPS response following a protein intake. Moreover, oral intake of intact whey protein results in a greater phenylalanine net balance across the limb than ingestion of the EAA or the non-essential AA portions of an identical bolus [24,25]. This finding suggests that whey protein stimulates muscle protein accretion through mechanisms that are beyond those of the EAA, such as an increased insulin secretion and, thus, an inhibited muscle protein breakdown (MPB) [26,27,28]. In addition, it has been suggested that a whole-meal feeding may favor protein accretion even more [29], however, it is beyond the scope of this review to discuss this further.

According to the “muscle full” concept the MPS will increase in a dose-response relation, as long as the total amount of EAA has not exceeded the maximal stimulating level (Figure 1). This is supported by the fact that consumption of 2.5–10 g EAA stimulates myofibrillar and sarcoplasmic MPS in a dose-dependent manner, while 20 and 40 g EAA fail to elicit any additional stimulation [30]. In agreement, a dose-dependent increase of mixed MPS has been observed with ingestion of protein following resistance exercise [31]. Moreover, a plateau was reached at 20 g protein (8.6 g EAA), with higher protein doses stimulating AA oxidation rather than MPS [31]. Additionally, ingestion of 30 and 90 g of high-quality beef protein did affect mixed MPS similarly in young men and women [32]. These results indicate that ingestion of approximately 20 g of protein (or 10 g of EAA) is sufficient to maximally stimulate MPS for a few hours both at rest and in the postexercise recovery period. In this regard, less is known about the maximal stimulating level in respect to the muscle protein balance. Due to the apparent separate and additive inhibitory effects of intracellular AA and insulin concentrations on MPB [33], it is possible that the muscle protein balance can benefit from even greater protein intakes than those observed to maximally stimulate MPS [29] (Figure 1). Hence, the muscle protein balance response to different levels of protein feeding should be an area for future research.

### 3.3. Consequences of Aging

**Figure 2:**
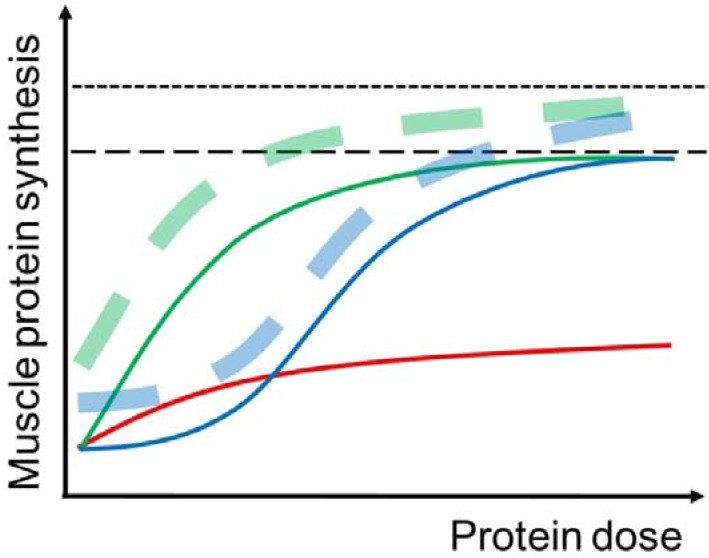
Illustration of the muscle protein synthetic (MPS) response to protein intake at rest (solid curves) and with prior completion of resistance exercise (dashed curves) in young (green curves) and elderly (blue curves) individuals. In the young, MPS is stimulated already at small doses (~5 g) of protein intake and the response reaches a plateau at approximately 20 g of protein intake. In the elderly, a higher amount of protein intake is necessary to simulate MPS and to obtain the maximal MPS response. The MPS response to protein intake can be enhanced by prior completion of resistance exercise, although the effect of resistance exercise is somewhat reduced in elderly compared to young individuals. Furthermore, the red curve illustrates that the MPS response to protein intake is reduced in immobilized muscle.

Even though the dietary protein digestion and absorption kinetics do not seem to be impaired in elderly compared to young individuals [34,35], the feeding-induced stimulation of MPS is blunted when small amounts of protein or the equivalent EAA content are ingested [23,30,36,37,38,39] (Figure 2). That is, ingestions corresponding to 20 g of protein or more have been shown to be necessary to stimulate MPS in elderly [38,39]. This phenomenon is termed “anabolic resistance” of elderly individuals. Additionally, the amount of protein ingestion that induces a maximal MPS response is higher in elderly than in young individuals [38]. More specific, mixed MPS was higher after ingestion of 35 g compared to 20 g whey protein in elderly [38], which was not observed in young individuals [31]. However, no additional stimulatory effect on myofibrillar MPS was observed by ingestion of 40 g compared to 20 g of whey protein in another recent study on elderly [39]. The discrepancy between those two studies may be that the MPS responses were measured in mixed [38] and myofibrillar [39] muscle proteins respectively. However, if so, other muscle protein fractions than the contractile proteins should respond differently to large amounts of protein intakes, which though does not seem to be the case for sarcoplasmic [18,30,40] and mitochondrial [41,42] muscle proteins. 

Despite the anabolic resistance in elderly, the maximal MPS response that can possibly be obtained by feeding seems to be similar in young and elderly individuals [32,34,35,43,44]. Additionally, an increased leucine intake has been shown to be an effective strategy to overcome the anabolic resistance to mixed meals [23,45] or smaller doses of EAA [37] in elderly individuals. Thus, it appears that a “threshold” of approximately 2 g of leucine must be surpassed in order to stimulate a rise in MPS in elderly individuals [36,37,39].

In addition to protein feeding, resistance exercise also elicits an impaired MPS response in elderly compared to young individuals [46,47,48] (Figure 2). The underlying reason for the “anabolic resistance” to protein feeding and resistance exercise in elderly is not known. However, studies indicate that impaired endothelial function and muscle anabolic signaling may be involved [30,49,50,51,52]. Specifically, an impaired endothelial function could limit blood flow and EAA delivery to the elderly muscles [50,52]. Additionally, the anabolic signaling of insulin and EAA is impaired in elderly muscles [30,49,51], which may be mediated by inflammatory activity [53] and hyperlipidemia [54]. Furthermore, the anabolic potential of insulin and EAA can be restored by anti-inflammatory treatment in elderly muscles [51,55,56]. Collectively, these findings might explain why the MPS response to lower doses of protein or EAA intakes is reduced in elderly and that higher intakes are needed to obtain the same response as in young individuals.

## 4. Effects of Activity Level and Proteins

### 4.1. Muscle Inactivity

It is known that muscle inactivity leads to loss of muscle mass [57,58,59,60,61,62,63,64,65,66,67], loss of muscle strength [57,58,59,60,61,62,63,64,68], and reduced muscle oxidative capacity [65,66] in humans. The loss of muscle tissue may be driven by a reduction in both the postabsorptive MPS [62,68,69,70] and the protein synthetic response to elevated EAA plasma concentrations [9,62,67]. However, signaling data indicate that also MPB could play a role in loss of muscle tissue during inactivity [57,66,70]. 

Whole-body [9], mixed muscle [67], and myofibrillar [62] protein synthetic response to elevated EAA concentrations has been shown to decrease following a period of inactivity (Figure 2). However, it has been shown that regular EAA ingestion during ≤4 week of bed rest can help maintain mixed MPS response to feeding in both young and elderly individuals [58,64]. Additionally, regular EAA ingestion prevented muscle loss in the young [58] but not in the elderly [64] individuals during the inactivity period. As discussed by Glover *et al.* [62], it seems unlikely that feeding of EAA supplements alone can prevent the detrimental effects of muscle inactivity, especially in elderly that may display a reduced anabolic sensitivity to feeding. Hence, more drastic interventions should be included to diminish muscle catabolism during periods of muscle inactivity. Recent findings indicate that anti-inflammatory treatment could have a beneficial effect on muscle gain during periods of training in elderly humans [71,72]. Likewise, during periods of muscle inactivity it could be hypothesized that combined intake of protein and anti-inflammatory treatment could diminish muscle loss.

With regard to changes in protein breakdown during inactivity, less is known. In the postabsorptive state a slight decrease in the whole-body protein breakdown rate has been reported [8,9], whereas no change in MPB measured indirectly after 28 days of bed rest and hypercortisolemia was reported [69,73]. In contrast to the latter findings in skeletal muscle, Brocca *et al.* demonstrated an increased amount of polyubiquitinated proteins after 24 days of bed rest alongside a significant loss of myofibrillar protein content [66]. Additionally, indications of increased macroautophagy activity were reported, which also could lead to muscle protein degradation [66]. These findings indicate that the ubiquitin proteasome system and the autophagy system are implicated in muscle atrophy following long time inactivity in humans. In support hereof, other studies indicate that the ubiquitin-dependent proteasome system may play a role in muscle protein degradation during inactivity in young humans [57,70], although this is not a universal finding [62]. However, the static intramuscular signaling measurements do not always reflect the muscle protein turnover measurements [26,62,70]. Hence, the genes involved the regulation of MBP only gives an indication of whether the degradative processes in the muscle are affected by disuse. So far the MPB has not been measured directly during periods of muscle inactivity, primarily due to lack of appropriate methods.

During periods of less or no muscle activity, the daily meal-induced periods of positive postprandial muscle protein balance will be attenuated and become insufficient to equalize the periods of negative muscle protein balance normally present between meals. Over time, this imbalance results in the seemingly inevitable loss of muscle mass. Therefore, we believe that normal daily muscle activity is necessary to maintain the sensitivity to nutrients in general and with special emphasis to AA and protein.

### 4.2. Muscle Exercise

Resistance exercise increases both MPS and MPB, but the MPS is stimulated to greater extent [74]. Therefore, the muscle protein net balance is improved, although it stays negative when no nutrition is ingested post-exercise [75]. The hypertrophic effect of resistance exercise *per se* is initiated shortly after completion of the muscle contractions [18,76] and persists for up to 1–2 days [77,78]. Furthermore, resistance exercise improves the hypertrophic potential of nutrients, especially AA and protein [75,79] (Figure 2), which is obtained by widening the limit of the “muscle full” concept allowing an incorporation of more AA into muscle protein during the postprandial period [18,39,80]. 

Probably due to methodological constraints, not much literature has measured MPS during exercise. During heavy resistance exercise MPS seems depressed [76], which is explained by energy conserving mechanisms directing intramuscular AA towards energy production rather than muscle adaptation [76,81,82]. Similarly, it was recently shown that during endurance exercise protein ingestion does not improve MPS compared to carbohydrate ingestion [83]. 

Recently, we reported that performance of light-load resistance exercise leads to prolonged utilization of circulating AA for MPS during continuous protein feeding [20]. Therefore, even brief light-load resistance exercise seems to be sufficient to expand the limitation of the “muscle full” concept, which we think is a very promising finding for individuals, who are unable to perform the more strenuous types of exercise (e.g., very frail elderly and hospitalized persons). Such a hypothesis is supported by longitudinal observations of muscle hypertrophy following 12 week of light-load resistance exercise [84]. The limits of the “muscle full” concept thus seem to be dependent on the activity level: where muscle inactivity narrows and muscle activity expands the limitations maybe in proportion to the severity of the intervention.

Based on the potential hypertrophic effect of even the light-load muscle contractions [20,80], endurance exercise might induce muscle hypertrophy as well. As compared to resistance exercise, endurance exercise differs in intensity, volume, and muscle fatigue. These factors have been shown to influence the potential hypertrophic effect of exercise [46,85,86]. Both in combination with repeated sprint interval exercise [87] and more prolonged endurance exercise [41,83,88,89] ingestion of protein or AA was recently shown to enhance MPS above that of exercise alone or exercise combined with carbohydrate feeding. However, since no protein-ingesting control group was included in these studies, it is presently unknown whether the observed effects were due to the protein feeding alone. Moreover, although greater type I and IIa myofiber sizes has been reported in young competitive runners than in young recreationally active individuals [90], the hypertrophic potential of endurance exercise has not been confirmed by others [42,91,92,93]. Additionally, it has been shown that endurance training can induce muscle hypertrophy in elderly individuals [94,95], although this is not a consistent finding [91,96,97,98]. Endurance exercise has been shown to decrease systemic inflammatory markers [99,100,101] and improve endothelial function [102,103,104] in elderly individuals. These effects could potentially counteract the “anabolic resistance” in elderly individuals, as discussed earlier. Therefore, endurance exercise may have a muscle-preserving effect in elderly individuals by counteracting “anabolic resistance”. But generally speaking, endurance exercise likely improves muscle quality more than muscle quantity [105,106,107]. In line with this, endurance training combined with immediate protein ingestion lead to improved muscle oxidative capacity, indicating that protein feeding improves the qualitative muscle adaptations to endurance training such as mitochondrial biogenesis [107].

## 5. Differences between Protein Types

It is commonly known that the quantity and quality (AA composition and digestibility) of a given protein intake have fundamental consequences for the changes in protein metabolism achieved by the intake. Since most studies in the field of muscle physiology have investigated the effects of milk proteins, the following discussion is limited to the two major types of milk protein: casein and whey. Both proteins have a favorable AA composition for stimulating MPS and, thus, we believe that the peripheral response is mainly determined by the proteins’ different digestive characteristics [108,109,110,111]: whey remains soluble in the stomach and is digested rapidly, but casein is converted to a solid clot in the gastric acidic environment and therefore the constituent AA are taken up slowly. 

At whole-body level, casein and whey proteins are shown to have different effects on protein metabolism in young resting subjects [108]. Generally, whey protein promotes high and short increments in protein synthesis and leucine oxidation [108]. In contrast, casein protein provokes a low but sustained increase in protein synthesis and a decrease in protein breakdown [108]. Importantly, ingestion of casein is shown to result in the largest whole-body protein gain [108]. However, because whey protein contains more leucine than casein, and because the meals were matched for the amount of leucine [108], the total amount of EAA was higher with casein than with whey feeding, which may have affected the protein metabolism. On the other hand, consumption of “fast” and “slow” AA/protein meals mimicking the whey and casein absorption rates, respectively, with identical AA compositions and nitrogen contents, revealed essentially the same alterations in whole-body protein metabolism in young resting men as observed with casein and whey proteins [109]. Hence, the digestion rate expressed as magnitude and duration of elevations in AA availability, and not the specific AA profile, is an independent regulating factor of the whole-body anabolic effects of casein and whey. 

Recently, sodium caseinate was found to have superior anabolic effects compared to whey in elderly COPD patients and healthy controls [112]. In that study, the caseinate maintained protein anabolism elevated both during and following the exercise bout. It was speculated whether the total amount of branched chain AA, and not solely leucine, was decisive for the response, since the content of branched chain AA and leucine was highest in caseinate and whey, respectively [112]. These findings are comparable to previous findings showing that milk soluble protein isolate (whey) was too rapidly digested and absorbed to maintain postprandial anabolism as compared to mixed milk proteins or casein [113]. Collectively, the utilization of protein seems to be best with a slower digested and absorbed protein type as compared to a fast type when determined at whole-body level both in the resting conditions and during recovery from exercise.

The effects of whey and casein have also been investigated at a skeletal muscle-specific level at rest and in relation to resistance exercise [114,115,116,117,118,119,120]. Whey and casein/caseinate have been found to have similar hypertrophic effects when measured for 0–6 h of recovery [114,116,117]. The net balance of phenylalanine across the limb was equal with ingestion of whey or casein proteins after resistance exercise [114] and, thus, it does not seem to be influenced by the different digestion rates of whey and casein. Likewise, similar myofibrillar MPS between whey and calcium caseinate have been observed during 6 h of recovery following heavy resistance exercise both in young [116] and elderly [117]. In contrast, micellar casein was found to be inferior to whey in other studies in young [115] and elderly [118,119]. However, except the study by Pennings *et al.* where the measuring period was 6 h [118], the measuring period following the intervention was only 3 h [115] and 4 h [119]. This fact might explain the overall discrepancy between the studies as whey seems to have a fast stimulating effect on MPS whereas the effect of casein/caseinate seems to be more moderate but prolonged. Therefore, the total hypertrophic response measured between different kinds of milk proteins may depend upon the duration of the measurement period. Recently, a very elegant study labeled whey and micellar casein with different isotopic tracers to show that the casein, when ingested together with whey (0.625 g/kg fat-free mass of both whey and casein and 0.9 g/kg fat-free mass of lactose), had a more sustained hypertrophic effect compared to whey during a total post meal period of 7 h [120].

To generalize, fast digested proteins of high quality seem to exert their stimulatory effect on MPS during the first 3 h, whereas slow digested proteins have similar positive effects on MPS when measured for 6–8 h. On a whole-body level and by estimating the total protein utilization and nitrogen retention, more slow digested proteins seem to be superior to faster types of protein. Therefore, the practical consideration should be that the kind of protein best suited to support muscle growth or muscle maintenance depends on the situation. Following exercise during day-time a fast protein would be most beneficial given that other meals are to be ingested within 2–3 h, but with exercise in the evening with no further main meal servings that day or as a bed-time supplement, a slow protein or simply milk (80% casein) could be speculated to be superior. For example, during night time, 40 g of casein has been shown to stimulate MPS in healthy young [121] and elderly [122] males. However, with regard to the slow digested proteins, there is a lack of studies investigating the dose-response relationship between the amount of protein ingested and the resultant MPS or net protein gain.

## 6. Long Term Effects of Nutrients and Resistance Exercise

Protein intake and muscle contractions are capable of improving muscle anabolism acutely as described in the previous sections. An important question, though, is how reliable and valid these acute responses are in regard to the long term adaptations to specific nutrient and/or training interventions. Factors related to the specific study designs and methodologies could help explain some of the discrepancies among the different studies: the measurements in the acute studies reflects the processes within the myofiber by measuring the incorporation of the AA tracer, whereas the longitudinal studies rely on somewhat crude techniques as whole body and whole muscle scans and in some studies cross-sectional area of single myofibers. Furthermore, the longitudinal training period may be too short to detect the accumulation of the rather small single hypertrophic events following each nutrient supplementation and/or exercise session.

Many acute studies conclude that the addition of protein ingestion in connection to resistance exercise has additive effects beyond the response of the exercise or the protein alone [18,75,123]. This additive effect has also been shown during longitudinal training studies [124,125,126,127]. Although long term training adaptations are mainly due to accumulation of the individual responses to each exercise session [128,129], other longitudinal training studies have shown that protein supplementation together with resistance exercise does not result in any extra gains in muscle mass [130,131,132,133,134]. Therefore, clear conclusions from single studies should be interpreted with caution. A recent meta-analysis by Cermak *et al.* has tried to decipher these conflicting results [135]. On the basis of pooled estimates, it was concluded that protein supplementation together with resistance-type exercise do have additive effects compared to exercise alone or with a placebo control, in both young and elderly [135]. 

The subjects enrolled in the different studies may vary a lot on training adaptive specific parameters, and the lack of homogeneity (study groups consisting of responders and non-responders) may have a major impact on the number of participants required to detect small differences. A recent example of the importance of the study group characteristics are two 24-week long resistance training and protein supplementation studies performed in elderly men and women (average age of 70 year) [136] and in frail elderly men and women (average age of 78 year) [137] by the same Dutch research group. The interventions in the two studies were identical, but it turned out that protein supplementation (15 g milk protein concentrate twice daily) had additive effects to exercise only in the group of frail elderly [136,137]. This illustrates the differences in training and protein derived muscle adaptability between different groups of individuals. 

Attempts to link acute responses with longitudinal adaptations in the same population and with the same intervention conducted by the same lab are reported in a few cases. In two papers from Wilkinson *et al.* and Hartman *et al.*, the authors demonstrate that a milk supplementation is superior to soy in combination with resistance exercise [126,138]. Acutely, it was shown that during 3 h of recovery from resistance exercise, MPS was increased more in the milk group than in the soy group [138]. In the 12-week heavy resistance training study, the most pronounced increases in fat- and bone-free mass and myofiber cross-sectional area were found in the milk group as compared to the soy and the carbohydrate groups [126]. 

Another approach trying to explain the sometimes conflicting findings in the literature was presented by Bosse and Dixon, who proposed the “protein spread” and “protein change” theory [139]. The protein spread theory illustrates that the difference between the protein supplementation intervention and control needs to be high, and the protein change theory illustrates that the protein supplementation intervention needs to differ with regard to the baseline (*i.e*., the normal daily protein consumption by the study participants). Their theories are based on a number of training and protein supplementation studies and advocates for a favorable dose-response effect of increasing protein intake relative to the basal level during periods with resistance exercise for gaining muscle mass and increasing muscle strength [139]. 

Presently, it seems to be beneficial both for young and elderly individuals to add extra protein to the diet during periods with resistance exercise. Having said that, it is open for discussion whether the protein supplement has a minor or a major additional effect to resistance exercise. Importantly, the potential extra benefit of protein supplementation are very likely highly dependent on the training and general nutritional status of the individual performing the training program. To get further insight into the mechanisms of muscle hypertrophy and the effect of protein and AA, there is a need to extend the duration of the muscle specific regulation of protein turnover and to combine acute responses with longitudinal data.

## 7. New Ways to Measure Muscle Protein Turnover

Translation of acute findings of skeletal muscle protein turnover to longitudinal training-based muscle hypertrophy is difficult. To exemplify, an enormous muscle accumulation would be expected if the long term adaptation of a high protein intake was to be foreseen based on the changes in the resting MPS reported within few hours after ingestion of protein in the fasted state. Furthermore, if short term measurements of underlying protein turnover rates of physiological conditions which results in distinct phenotypes do not reveal any differences, it may be argued that the lack of differences relies on a statistical type II error. Methodologically, this means that the inherent differences may be too small for the short term tracer-applications to detect them. The “golden standard” of stable isotope tracer techniques generally used in acute exercise and nutrition studies have to be performed under very standardized settings, which are often incomparable with a normal daily pattern of physical activity and nutrition. The standardized settings in these acute studies often include overnight fasting and supine resting except of short and controlled instances of nutritional and/or physical activity interventions. In longitudinal training studies the participants’ daily living inevitable has a major effect on the single acute responses and long-term adaptations. Therefore, to investigate the significance of acute fluctuations in the protein turnover rates more prolonged and daily-life measurements are requested [140]. 

Two tracer approaches allow to go beyond the standardized, short term protein turnover measurements and in to a prolonged real-life situation: (1) the ingestion of intrinsically labeled proteins as route of administering the AA tracer [141] and (2) administration of deuterated water as a label-donor to AA that are subsequently incorporated into protein and can be traced for measuring both protein synthesis and breakdown [142,143]. 

The use of intrinsically labeled proteins to administer the AA tracer has been shown useful in few studies to determine the fractional synthesis of body proteins [34,35,141,144]. The advantage of using this approach is that the AA kinetics can be investigated under real-life circumstances [145]. Despite its limited tracer content, ingestion of intrinsically labeled proteins can maintain the precursor enrichment sufficiently elevated, over a prolonged period of time, to allow detection of significant incorporation in to, e.g., slow turnover muscle proteins [141,144]. This setting can be used to study eating and physical activity patterns in free-living individuals as well as under circumstances where intravenous tracer infusion is not possible (e.g., hospitalized patients). This approach is mainly suitable for single day measurements where intake of protein can be distributed throughout the day. During night-time, sustained tracer abundance can be maintained by protein ingestion just before bed time and/or during night, although this would violate the “normal life patterns” [121,122]. Alternatively, night-time measures could be conducted by starting up an intravenous tracer infusion before bedtime. This, though, would require that the steady state tracer enrichment from the protein intake is known. The use of intrinsically labeled proteins has also proved very valuable to trace the uptake of ingested AA by the splanchnic bed [34,146], giving more valid results than when assessed with free AA tracers [145,147]. Furthermore, it has proved to reveal novel insights into the fate of the food-derived AA once they appear in the circulation [108,146]. 

If more prolonged measurements of protein synthesis rates are to be measured, the administration of deuterated water is preferable. The principle of this approach is different from the classic use of stable isotopically labeled AA [148]. Briefly, the deuterated water mixes quickly (few hours in humans) into all water pools in the body and exchanges deuterium with, e.g., alanine at up to four sites, hence labeling alanine at positions 2,3,3,3-^2^H_4_-alanine [142,149]. Once equilibrated, this exchange is not markedly affected by feeding or activity [149], probably due to the enormous pool of body water buffering the minor disturbances in alanine enrichment from exogenous uptake and de novo synthesis of alanine. Furthermore, the enrichment of the AA precursor for protein synthesis can be kept constant by repeated boluses of deuterated water [142,143]. Dependent on the timely extend of this approach, the fractional synthesis rate can be calculated over short time intervals using the linear incorporation model [150] or over prolonged time periods using the exponential rise, where the recirculation of the tracers must be taken into account [142,143,151]. The prolonged data has been reported in human *in vivo* settings [107] showing promising features.

As the last inevitable comments, only the mixed or myofibrillar MPS is measured in most studies. Therefore, these studies do not give any information on MPB or the net muscle protein balance, and the possibility of a poor prediction of the long term outcome may be present based on these acute changes in MPS. Comparable methods to measure both protein synthesis and breakdown are requested. Recently, a novel method was presented [152], which seems useful and can be applied using deuterated water [153].

## 8. Conclusions

Ingestion of protein leads to muscle protein anabolism. However, the absolute amount of AA that can be incorporated into human contractile muscle protein during hyperaminoacidemia is limited. This quantitative limitation, a phenomenon termed the “muscle full” concept, can be modulated by physical activity/training; where muscle inactivity narrows and muscle activity expands the limitations. The protein digestibility is decisive for the appearance and utilization of AA and hence, an important determinant for the response in skeletal muscle. Both “slow” and “fast” proteins inhere advantages and their specific use is dependent on the daily life situations in which they are ingested. To further explore the physiological significance of interventions including nutrient intake and exercise, new approaches that link the acute muscle response and the long-muscle term adaptation should be applied. 
